# The effect of sumatriptan on cephalic arteries - 3T MR-angiography study in healthy volunteers

**DOI:** 10.1186/1129-2377-1-S14-P210

**Published:** 2013-02-21

**Authors:** FM Amin, MS Asghar, JW Ravneberg, PJH de Koning, HBW Larsson, J Olesen, M Ashina

**Affiliations:** 1 Danish Headache Center & Dept. of Neurology, Denmark; 2 Division of Image Processing, Netherlands; 3 Functional Imaging Unit, Department of Diagnostic, Glostrup Hospital, Denmark

## Introduction

The triptans, serotonin receptor B/D agonists, are the mainstay in the acute treatment of migraine[1]. Sumatriptan, the first triptan, was originally developed as a cranial vasoconstrictor by acting on the 5-HT1B/1D receptors in cephalic vessels[2]. However, several modes of action as well as multiple sites of action have been proposed.[3]

## Objectives

To explore a possible differential effect of sumatriptan on extra- versus intracerebral arteries, we examined the superficial temporal (STA), middle meningeal (MMA), extracranial internal carotid (ICAextra), intracranial internal carotid (ICAintra), middle cerebral (MCA) and basilar artery (BA).

## Methods

The arterial circumference were recorded by high resolution magnetic resonance angiography before and after subcutaneous sumatriptan injection (6 mg) in 18 healthy volunteers.

## Results

We found significant constrictions of MMA (16.5%), STA (16.4%), ICAextra (15.2%), MCA (5.5%) and BA (2.1%) (p ≤ 0.012). ICAintra changed by 1.8% (p = 0.179). Analyses of the relative changes between the intracerebral and extracerebral arteries revealed significantly larger constriction of the extracerebral than of the intracerebral (MCA, BA, ICAintra) arteries (p < 0.000001).

## Conclusion

Sumatriptan constricts more extracerebral than intracerebral arteries. We suggest that sumatriptan exerts its anti-migraine action outside the blood-brain barrier by constricting the extracerebral arteries and blocking the nociceptive inputs from sensory afferents.

## Conflict of interest

JO has received grants/research support from, has been a consultant/scientific adviser for, and has been in the speakers’ bureau of Allergan Inc, AstraZeneca Pharmaceuticals LP, Boehringer Ingelheim, Eli Lilly, GlaxoSmithKline, Janssen Pharmaceutical Products, Lundbeck, Merck and Pfizer. MA has received grant support/honoraria for lecturing from Merck, honoraria for lecturing from Pfizer, GlaxoSmithKline, Norpharma and AstraZeneca, is a consultant/scientific adviser for Merck and Allergan. FA has received honoraria for lecturing from Allergan.
